# Supplementary material for: Emerging Technologies for Dentin Caries Detection—A Systematic Review and Meta-Analysis
Source: J Clin Med. 2022 Jan 28;11(3):674. doi: 10.3390/jcm11030674 (PMC8837049; doi:10.3390/jcm11030674)
Supplement: Supplementary file 1 [file jcm-11-00674-s001.zip › sup/CariesTech-Supplementary2.pdf]

# Supplementary material

## Additional meta-analytical results

**Table S1**. Risk of bias assessments for *in vivo* studies

| | *Signaling questions* | | | | | | | | | | | | | | | |
|---|---|---|---|---|---|---|---|---|---|---|---|---|---|---|---|---|
| | Selection bias | | | | Index test bias | | | Reference test bias | | | | Verification bias | | | Outcome bias | |
| | Patient selection | Teeth selection | Caries Spectrum | Sample size | Test Criteria | Blinding bias | Calibration bias | Test Criteria | Blinding bias | Calibration bias | Incorporation bias | Partial ver. bias | Differential ver. bias | Bias in the Analysis | Validity bias | Reproducibility bias |
| *Castilho 2016* | - | + | + | - | + | + | + | + | + | - | + | + | + | + | + | + |
| *Diniz 2012* | - | - | - | - | + | + | + | + | + | - | + | + | + | + | + | ? |
| *Dundar 2020* | ? | ? | - | - | + | + | - | - | + | - | + | + | + | + | + | ? |
| *Jablonski-Momemi 2014* | ? | - | + | + | + | + | + | + | + | - | + | + | + | + | + | + |
| *Laitala 2017* | - | + | - | + | + | + | + | - | + | - | + | + | + | + | + | - |
| *Litzenburger 2021* | ? | + | - | + | + | + | + | - | + | - | + | + | + | + | + | + |
| *Ozkan 2017* | - | ? | - | + | + | - | + | + | - | - | + | + | + | + | + | + |
| *Shimada 2014* | ? | ? | ? | - | + | + | - | - | + | - | + | + | + | + | + | - |
| *Tassoker 2019* | - | - | + | - | + | + | - | + | + | - | + | + | + | + | + | - |

**Table S2**. Risk of bias assessments for *in vitro* studies

| | *Signaling questions* | | | | | | | | | | | | | | | |
|---|---|---|---|---|---|---|---|---|---|---|---|---|---|---|---|---|
| | **Selection bias** | | | | **Index test bias** | | | **Reference test bias** | | | | **Verification bias** | | | **Outcome bias** | |
| | Patient selection | Teeth | Caries | Sample size | Test Criteria | Blinding bias | Calibration | Test Criteria | Blinding bias | Calibration | Incorporation | Partial ver. | Differential | Bias in the | Validity bias | Reproducibili |
| *Achilleos 2013* | x | - | - | - | + | + | + | + | + | - | + | + | + | + | + | + |
| *Aktan 2012* | x | + | + | - | + | + | + | + | + | + | + | + | + | + | + | ? |
| *Astvaldsdottir 2012* | x | - | ? | - | + | + | ? | + | + | ? | + | + | + | + | + | + |
| *Bizhang 2016* | x | + | + | + | + | + | - | - | + | - | + | + | + | + | + | - |
| *Bozdemir 2016* | x | - | ? | - | + | - | - | + | + | - | + | + | + | + | + | ? |
| *Bussaneli 2015* | x | - | - | - | + | + | - | + | - | - | + | + | + | + | ? | + |
| *De Paula 2011* | x | + | - | - | + | + | + | + | + | - | + | + | + | + | + | + |
| *De Souza 2014* | x | - | + | + | + | + | - | + | + | - | + | + | + | + | + | + |
| *Gomez 2013* | x | - | - | - | + | + | - | + | + | - | + | + | + | + | + | ? |
| *Jablonski-Momemi 2011* | x | - | - | - | + | + | + | + | + | - | + | + | + | + | + | + |
| *Jablonski-Momemi 2012a* | x | - | - | - | + | + | + | + | + | - | + | + | + | + | + | - |
| *Jablonski-Momemi 2012b* | x | + | - | - | + | + | + | + | + | - | + | + | + | + | + | - |
| *Jablonski-Momemi 2013* | x | + | - | - | + | + | + | + | + | - | + | + | + | + | + | - |
| *Jallad 2015* | x | - | ? | - | + | + | + | + | + | - | + | + | + | + | ? | + |
| *Ko 2015* | x | ? | - | - | + | + | - | + | - | - | + | + | + | + | + | ? |
| *Lederer 2019* | x | ? | ? | - | + | + | + | + | + | - | + | + | + | + | + | + |
| *Luong 2020* | x | + | ? | + | + | + | + | - | + | - | + | + | + | + | + | ? |
| *Luczaj-Cepowciz 2019* | x | - | - | + | + | + | + | + | + | - | + | + | + | + | + | + |
| *Marczuk-Kolada 2020* | x | - | - | - | + | + | + | + | + | - | + | + | + | + | + | + |
| *Mortensen 2014* | x | + | - | - | + | + | + | + | + | - | + | + | + | + | + | + |
| *Nakagawa 2013* | x | + | ? | - | + | + | + | + | + | - | + | + | + | + | + | + |
| *Neuhaus 2015* | x | ? | ? | ? | + | + | ? | + | + | - | + | + | + | + | + | + |
| *Peycheva 2016* | x | ? | - | - | + | ? | - | + | + | - | + | + | + | + | + | + |
| *Rodrigues 2011* | x | + | ? | - | + | + | + | + | + | - | + | + | + | + | + | + |
| *Seremidi 2012* | x | - | + | + | + | + | + | + | + | + | + | + | + | + | + | ? |
| *Tassoker 2019* | x | - | + | - | + | + | - | + | + | - | + | + | + | + | + | - |

**Table S3**. Descriptive summary statistics for all studies included in meta-analysis

| Technology | Study ID | Sensitivity and 95%CI | | | Specificity and 95%CI | | |
|---|---|---|---|---|---|---|---|
| | | sens | 2.50% | 97.50% | spec | 2.50% | 97.50% |
| DIAGNOdent 2095 | de Paula 2011[50] | 0.447 | 0.252 | 0.66 | 0.744 | 0.594 | 0.852 |
| DIAGNOdent 2095 | Jablonski-Momemi 2012a[54] | 0.5 | 0.295 | 0.705 | 0.864 | 0.765 | 0.926 |
| DIAGNOdent 2095 | Rodrigues 2011[63] | 0.625 | 0.441 | 0.779 | 0.856 | 0.758 | 0.919 |
| DIAGNOdent 2095 | Diniz 2012[52] | 0.804 | 0.624 | 0.91 | 0.766 | 0.661 | 0.846 |
| DIAGNOdent 2095 | Castilho 2016[49] | 0.875 | 0.396 | 0.987 | 0.841 | 0.701 | 0.923 |

DIAGNOdent 2095
Test for equality of sensitivities: Chi-square = 8.722 (df = 4), p-value = 0.0684
Test for equality of specificities: Chi -square = 4.818 (df = 4), p-value = 0.307

| Technology | Study ID | Sensitivity and 95%CI | | | Specificity and 95%CI | | |
|---|---|---|---|---|---|---|---|
| | | sens | 2.50% | 97.50% | spec | 2.50% | 97.50% |
| DIAGNOdent pen | Bizhang 2016[47] | 0.795 | 0.703 | 0.864 | 0.966 | 0.935 | 0.982 |
| DIAGNOdent pen | De Souza 2014[51] | 0.931 | 0.8 | 0.978 | 0.899 | 0.851 | 0.932 |
| DIAGNOdent pen | Neuhaus 2015[60] | 0.413 | 0.239 | 0.612 | 0.84 | 0.755 | 0.9 |
| DIAGNOdent pen | Achilleos 2013[45] | 0.643 | 0.303 | 0.882 | 0.591 | 0.422 | 0.74 |
| DIAGNOdent pen | Aktan 2012[46] | 0.946 | 0.798 | 0.988 | 0.519 | 0.424 | 0.613 |
| DIAGNOdent pen | Bussaneli 2015[48] | 0.675 | 0.457 | 0.837 | 0.82 | 0.718 | 0.891 |
| DIAGNOdent pen | Jablonski-Momemi 2012b[55] | 0.806 | 0.577 | 0.926 | 0.583 | 0.463 | 0.694 |
| DIAGNOdent pen | Peycheva 2016[62] | 0.885 | 0.621 | 0.973 | 0.691 | 0.523 | 0.82 |
| DIAGNOdent pen | Rodrigues 2011 [63] | 0.625 | 0.441 | 0.779 | 0.866 | 0.768 | 0.927 |
| DIAGNOdent pen | Seremidi 2012[64] | 0.548 | 0.344 | 0.736 | 0.858 | 0.77 | 0.916 |
| DIAGNOdent pen | Tassoker 2019 [65] | 0.595 | 0.387 | 0.774 | 0.923 | 0.837 | 0.965 |
| DIAGNOdent pen | Mortensen 2014[59] | 0.25 | 0.144 | 0.398 | 0.825 | 0.71 | 0.901 |
| DIAGNOdent pen | Luczaj-Cepowciz 2019[57] | 0.441 | 0.239 | 0.665 | 0.864 | 0.709 | 0.943 |
| DIAGNOdent pen | Marczuk-Kolada 2020[58] | 0.712 | 0.519 | 0.849 | 0.805 | 0.731 | 0.863 |
| DIAGNOdent pen | Diniz 2012[52] | 0.839 | 0.664 | 0.932 | 0.703 | 0.594 | 0.792 |
| DIAGNOdent pen | Litzenburger 2021[56] | 0.75 | 0.584 | 0.865 | 0.852 | 0.745 | 0.919 |
| DIAGNOdent pen | Tassoker 2019 [65] | 0.5 | 0.303 | 0.697 | 0.768 | 0.657 | 0.851 |
| DIAGNOdent pen | Dundar 2020 [53] | 0.808 | 0.724 | 0.872 | 0.852 | 0.827 | 0.874 |
| DIAGNOdent pen | Ozkan 2017[61] | 0.602 | 0.524 | 0.675 | 0.25 | 0.06 | 0.635 |

DIAGNOdent pen
Test for equality of sensitivities: Chi-square = 103.514 (df = 18), p-value < 0.0001
Test for equality of specificities: Chi -square = 191.971 (df = 18), p-value < 0.0001

| Technology | Study ID | Sensitivity and 95%CI | | | Specificity and 95%CI | | |
|---|---|---|---|---|---|---|---|
| | | sens | 2.50% | 97.50% | spec | 2.50% | 97.50% |
| VistaProof manufacturer cut-off | Achilleos 2013[45] | 0.5 | 0.202 | 0.798 | 0.809 | 0.648 | 0.907 |
| VistaProof manufacturer cut-off | Jablonski-Momemi 2011[66] | 0.104 | 0.033 | 0.285 | 0.967 | 0.9 | 0.99 |
| VistaProof manufacturer cut-off | Seremidi 2012[64] | 0.024 | 0.002 | 0.192 | 0.949 | 0.881 | 0.979 |
| VistaProof optimal cut-off | Jablonski-Momemi 2011[66] | 0.854 | 0.665 | 0.945 | 0.664 | 0.553 | 0.76 |

| | | | | | | | |
|---|---|---|---|---|---|---|---|
| VistaProof optimal cut-off | Jablonski-Momemi 2012b[55] | 0.806 | 0.577 | 0.926 | 0.583 | 0.463 | 0.694 |
| VistaProof optimal cut-off | Jablonski-Momemi 2013[67] | 0.894 | 0.782 | 0.952 | 0.777 | 0.639 | 0.872 |
| VistaProof optimal cut-off | Rodrigues 2011[63] | 0.946 | 0.798 | 0.988 | 0.697 | 0.582 | 0.792 |
| VistaProof optimal cut-off | Seremidi 2012[64] | 0.929 | 0.741 | 0.983 | 0.676 | 0.573 | 0.765 |
| VistaProof manufacturer cut-off | Jablonski-Momemi 2014[68] | 0.26 | 0.129 | 0.455 | 0.977 | 0.952 | 0.989 |

VistaProof
Test for equality of sensitivities: Chi-square = 121.086 (df = 8), p-value < 0.0001
Test for equality of specificities: Chi -square = 135.499 (df = 8), p-value < 0.0001

| Technology | Study ID | Sensitivity and 95%CI | | | Specificity and 95%CI | | |
|---|---|---|---|---|---|---|---|
| | | **sens** | **2.50%** | **97.50%** | **spec** | **2.50%** | **97.50%** |
| VistaCam iX manufacturer cut-off | Jablonski-Momemi 2012a[54] | 0.203 | 0.104 | 0.357 | 0.989 | 0.902 | 0.999 |
| VistaCam iX manufacturer cut-off | Marczuk-Kolada 2020[58] | 0.146 | 0.055 | 0.335 | 0.967 | 0.923 | 0.987 |
| VistaCam iX optimal cut-off | Jablonski-Momemi 2013[67] | 0.856 | 0.736 | 0.927 | 0.67 | 0.527 | 0.787 |

VistaCam iX
Test for equality of sensitivities: Chi-square = 51.43 (df = 2), p-value < 0.0001
Test for equality of specificities: Chi -square = 42.333 (df = 2), p-value < 0.0001

| Technology | Study ID | Sensitivity and 95%CI | | | Specificity and 95%CI | | |
|---|---|---|---|---|---|---|---|
| | | **sens** | **2.50%** | **97.50%** | **spec** | **2.50%** | **97.50%** |
| DIAGNOcam | Lederer 2019[69] | 0.806 | 0.577 | 0.926 | 0.976 | 0.925 | 0.993 |
| DIAGNOcam | Ozkan 2017[61] | 0.602 | 0.524 | 0.675 | 0.25 | 0.06 | 0.635 |
| DIAGNOcam | Dundar 2020[53] | 0.986 | 0.942 | 0.997 | 0.941 | 0.923 | 0.955 |
| DIAGNOcam | Litzenburger 2021[56] | 0.956 | 0.829 | 0.99 | 0.664 | 0.542 | 0.768 |

DIAGNOcam
Test for equality of sensitivities: Chi-square = 64.189 (df = 3), p-value < 0.0001
Test for equality of specificities: Chi -square = 106.281 (df = 3), p-value < 0.0001

| Technology | Study ID | Sensitivity and 95%CI | | | Specificity and 95%CI | | |
|---|---|---|---|---|---|---|---|
| | | **sens** | **2.50%** | **97.50%** | **spec** | **2.50%** | **97.50%** |
| Midwest | Bozdemir 2016[70] | 0.388 | 0.253 | 0.542 | 0.919 | 0.856 | 0.956 |
| Midwest | Neuhaus 2015[60] | 0.109 | 0.034 | 0.295 | 0.954 | 0.892 | 0.981 |
| Midwest | Rodrigues 2011[63] | 0.696 | 0.511 | 0.834 | 0.894 | 0.802 | 0.947 |
| Midwest | Aktan 2012[46] | 0.518 | 0.342 | 0.689 | 0.869 | 0.79 | 0.921 |
| Midwest | Dundar 2020[53] | 0.565 | 0.471 | 0.655 | 0.808 | 0.781 | 0.833 |

Midwest
Test for equality of sensitivities: Chi-square = 23.168 (df = 4), p-value < 0.0001
Test for equality of specificities: Chi -square = 25.131 (df = 4), p-value < 0.0001

| Technology | Study ID | Sensitivity and 95%CI | | | Specificity and 95%CI | | |
|---|---|---|---|---|---|---|---|
| | | **sens** | **2.50%** | **97.50%** | **spec** | **2.50%** | **97.50%** |
| OCT 2D | Gomez 2013[71] | 0.33 | 0.213 | 0.473 | 0.978 | 0.909 | 0.995 |
| OCT 3D | Luong 2020[72] | 0.792 | 0.509 | 0.933 | 0.76 | 0.628 | 0.855 |
| OCT 2D | Nakagawa 2013[73] | 0.938 | 0.818 | 0.98 | 0.949 | 0.883 | 0.979 |
| OCT 2D | Shimada 2014[74] | 0.868 | 0.716 | 0.945 | 0.917 | 0.813 | 0.965 |

| |
|---|
| OCT<br>Test for equality of sensitivities: Chi-square = 47.655 (df = 3), p-value < 0.0001<br>Test for equality of specificities: Chi -square = 21.574 (df = 3), p-value < 0.0001 |

| Technology | Study ID | Sensitivity and 95%CI | | | Specificity and 95%CI | | |
|---|---|---|---|---|---|---|---|
| | | sens | 2.50% | 97.50% | spec | 2.50% | 97.50% |
| FOTI | Astvaldsdottir 2012[75] | 0.344 | 0.163 | 0.585 | 0.946 | 0.875 | 0.978 |
| FOTI | Gomez 2013[71] | 0.777 | 0.639 | 0.872 | 0.918 | 0.827 | 0.963 |
| FOTI | Laitala 2017[76] | 0.462 | 0.375 | 0.552 | 0.932 | 0.92 | 0.942 |
| FOTI<br>Test for equality of sensitivities: Chi-square = 16.32 (df = 2), p-value < 0.0001<br>Test for equality of specificities: Chi -square = 0.355 (df = 2), p-value = 0.765 | | | | | | | |

| Technology | Study ID | Sensitivity and 95%CI | | | Specificity and 95%CI | | |
|---|---|---|---|---|---|---|---|
| | | sens | 2.50% | 97.50% | spec | 2.50% | 97.50% |
| QLF Inspektor Pro | Bussaneli 2015[48] | 0.694 | 0.464 | 0.856 | 0.825 | 0.725 | 0.894 |
| QLF Inspektor Pro | Gomez 2013[71] | 0.862 | 0.736 | 0.933 | 0.799 | 0.688 | 0.877 |
| QLF Inspektor Pro | Jallad 2015[78] | 0.875 | 0.73 | 0.948 | 0.596 | 0.407 | 0.76 |
| QLF Inspektor Pro | Ko 2015[77] | 0.633 | 0.387 | 0.825 | 0.872 | 0.783 | 0.928 |
| QLF Inspektor Pro<br>Test for equality of sensitivities: Chi-square = 6.304 (df = 3), p-value = 0.0977<br>Test for equality of specificities: Chi -square = 3.84 (df = 3), p-value = 0.02 | | | | | | | |

| Technology | Study ID | Sensitivity and 95%CI | | | Specificity and 95%CI | | |
|---|---|---|---|---|---|---|---|
| | | sens | 2.50% | 97.50% | spec | 2.50% | 97.50% |
| LIF | Gomez 2013[71] | 0.819 | 0.687 | 0.904 | 0.858 | 0.755 | 0.922 |
| LIF | Peycheva 2016[62] | 0.577 | 0.323 | 0.796 | 0.985 | 0.874 | 0.998 |
| LIF<br>Test for equality of sensitivities: Chi-square = 2.123 (df = 1), p-value = 0.145<br>Test for equality of specificities: Chi -square = 2.783 (df = 1), p-value = 0.095 | | | | | | | |

| Technology | Study ID | Sensitivity and 95%CI | | | Specificity and 95%CI | | |
|---|---|---|---|---|---|---|---|
| | | sens | 2.50% | 97.50% | spec | 2.50% | 97.50% |
| ACIS | Mortensen 2014[59] | 0.074 | 0.023 | 0.211 | 0.978 | 0.91 | 0.995 |
| ACIS | Litzenburger 2021[56] | 0.632 | 0.465 | 0.773 | 0.945 | 0.86 | 0.98 |
| ACIS<br>Test for equality of sensitivities: Chi-square = 21.622 (df = 1), p-value < 0.0001<br>Test for equality of specificities: Chi -square = 0.326 (df = 1), p-value =0.568 | | | | | | | |

| Technology | Study ID | Sensitivity and 95%CI | | | Specificity and 95%CI | | |
|---|---|---|---|---|---|---|---|
| | | sens | 2.50% | 97.50% | spec | 2.50% | 97.50% |
| PTR-LUM | Jallad 2015[78] | 0.847 | 0.697 | 0.93 | 0.442 | 0.271 | 0.628 |

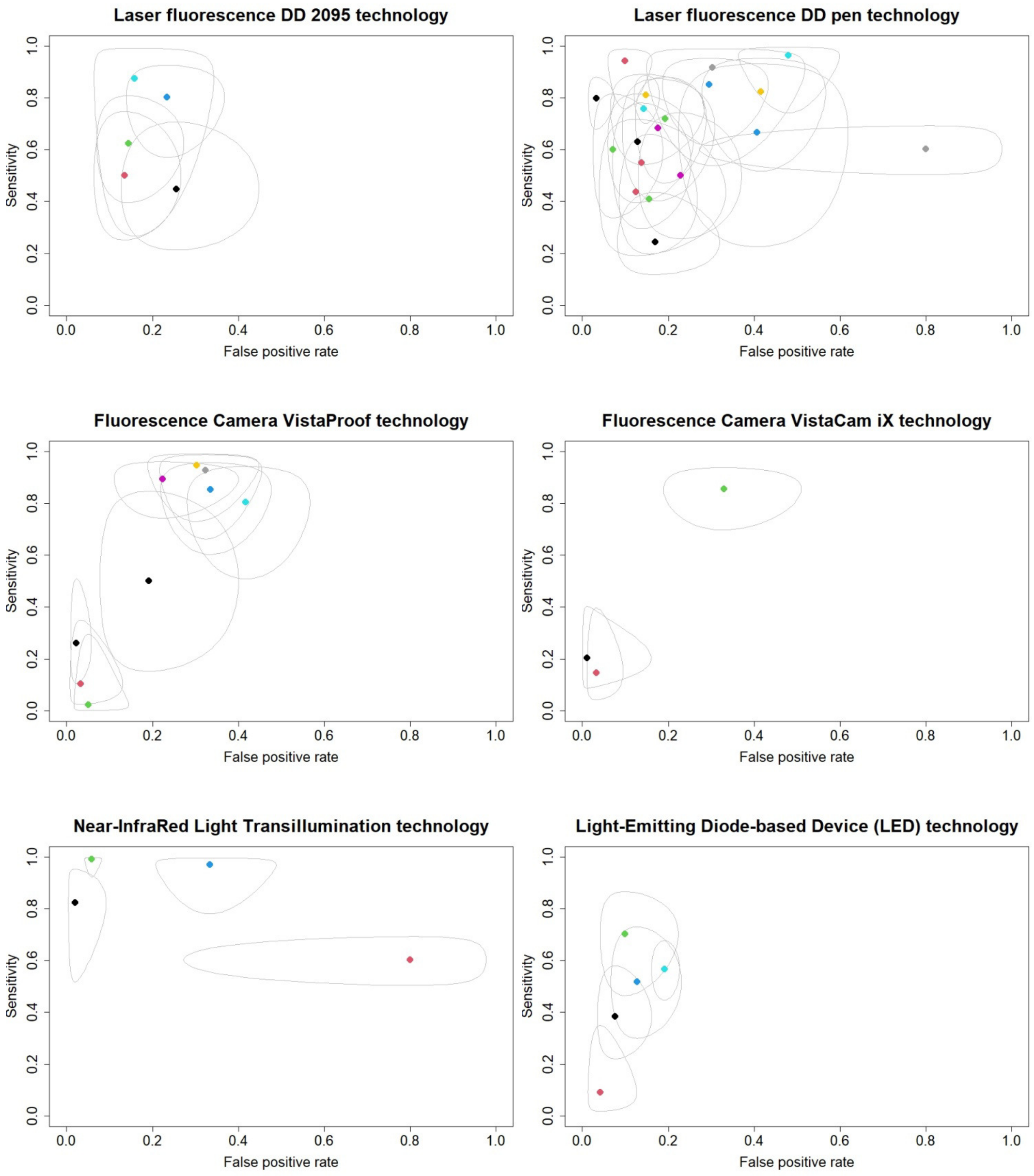


**Figure S1a.** ROC ellipses plots for all technologies included in meta-analysis (1/2). The points show the pairs of sensitivity and specificity for each study, and the curves plot their respective confidence regions on ROC space.

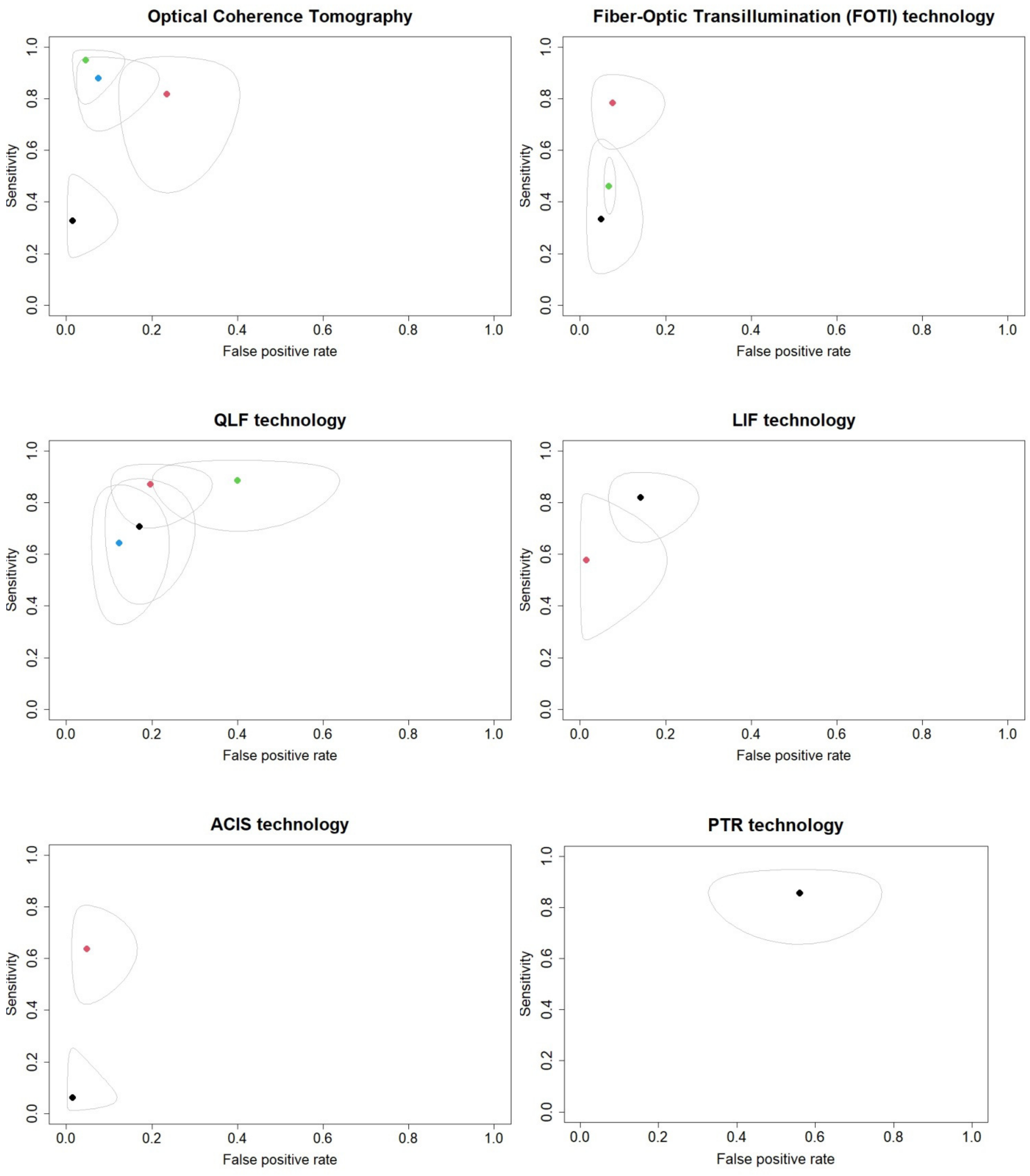


**Figure S1b.** ROC ellipses plots for all technologies included in meta-analysis (2/2). The points show the pairs of sensitivity and specificity for each study, and the curves plot their respective confidence regions on ROC space.
